# Significant association between clinical characteristics and changes in peripheral immuno-phenotype in large vessel vasculitis

**DOI:** 10.1186/s13075-019-2068-7

**Published:** 2019-12-30

**Authors:** Kotaro Matsumoto, Katsuya Suzuki, Keiko Yoshimoto, Noriyasu Seki, Hideto Tsujimoto, Kenji Chiba, Tsutomu Takeuchi

**Affiliations:** 1 Division of Rheumatology, Department of Internal Medicine, Keio University School of Medicine, 35 Shinanomachi, Shinjuku-ku, Tokyo, Japan; 2 Clinical and Translational Research Center, Keio University Hospital, 35 Shinanomachi, Shinjuku-ku, Tokyo, Japan; 3 Mitsubishi Tanabe Pharma Corporation, 1000, Kamoshida-cho, Aoba-ku, Yokohama, Kanagawa Japan

**Keywords:** Large vessel vasculitis, Giant cell arteritis, Takayasu arteritis, Immuno-phenotyping, Flow cytometry

## Abstract

**Background:**

Large vessel vasculitis (LVV) is a type of vasculitis characterized by granulomatous inflammation of medium- and large-sized arteries. Clinical assessment of acute phase reactants has been conventionally used to diagnose and monitor diseases; however, accurate assessment of vascular disease activity status can be difficult. In this study, we investigated comprehensive immuno-phenotyping to explore useful biomarkers associated with clinical characteristics.

**Methods:**

Consecutive patients with newly diagnosed LVV who visited our institution between May 2016 and May 2019 were enrolled. The number of circulating T cells, B cells, natural killer cells, dendritic cells, monocytes, and granulocytes was examined and chronologically followed. Baseline and time-course changes in immuno-phenotyping associated with disease activity were assessed.

**Results:**

Comprehensive immuno-phenotyping data from 90 samples from each of 20 patients with LVV were compared with those from healthy controls (HCs). The number of helper T (Th), follicular helper T (Tfh), CD8^+^ T, CD14^++^ CD16^+^ monocytes, and neutrophils were higher in patients with giant cell arteritis (GCA) and/or Takayasu arteritis (TAK) than in HCs. Among them, the number of CD8^+^ T and CD8^+^ Tem were higher in patients with TAK than in GCA. Notably, memory CD4^+^ and CD8^+^ T cells in patients with TAK remained high even in the remission phase. Further analysis revealed that the number of Th1, Th17, and Tfh cells was associated with disease relapse in GCA and TAK and that the number of CD8^+^ T cells was associated with relapse in TAK. Th1, Th17, and Tfh cells decreased after treatment with biologic agents, while CD8^+^ T cells did not.

**Conclusions:**

Our results from peripheral immuno-phenotyping analysis indicate that the numbers of Th and Tfh cells changed along with the disease condition in both GCA and TAK, while that of CD8^+^ T cells did not, especially in TAK. Treatment with biologic agents decreased the proportion of Th and Tfh cells, but not CD8^+^ T cells, in the patients. Chronological immuno-phenotyping data explained the difference in therapeutic response, such as reactivities against biologics, between GCA and TAK.

## Background

Large vessel vasculitis (LVV) is a type of vasculitis characterized by granulomatous inflammation of medium- and large-sized arteries [1–3]. According to the Chapel Hill Consensus Conference 2012 definition [4], giant cell arteritis (GCA) and Takayasu arteritis (TAK) constitute different types of primary LVV.

Recent clinical trials for LVV with tocilizumab (TCZ) have revealed that therapies targeting interleukin (IL)-6 are effective for reducing relapse and glucocorticoid (GC) dose in patients [5–7]. Also, tumor necrosis factor (TNF)-α inhibition therapies such as infliximab (IFX), etanercept, and adalimumab is effective for TAK [8, 9], and T lymphocyte-targeted therapy represented by abatacept is effective for GCA [10]. While targeted therapies have been successful, a complete picture of how immune cell profiles reflect the pathophysiology of LVV is currently lacking. In particular, differences in the baseline immunological profile between GCA and TAK and changes during treatment with GC and/or biologics are not well described.

LVV are intractable rare disease entities with a high relapse rate. Disease progression in asymptomatic patients is an important issue in the clinical management of LVV. There is a strong expectation that useful biomarkers may be present in the peripheral blood. We therefore aimed to identify the immunological characteristics of LVV and their clinical significance by comprehensively examining the immuno-phenotypes of LVV patients.

## Patients and methods

### Patients and healthy controls

Patients with newly diagnosed LVV who visited Keio University Hospital and fulfilled the American College of Rheumatology criteria for GCA [11] and TAK [12] between May 2016 and May 2019 were consecutively enrolled. Patients with secondary LVV that could mimic GCA/TAK (for example, Cogan syndrome, sarcoidosis, Kawasaki disease, Behçet disease, IgG4-related disease, syphilis, tuberculosis, Ehlers-Danlos syndrome, Marfan syndrome, and neurofibromatosis) were excluded based on the medical chart at screening. We confirmed that the healthy controls (HCs) did not have an autoimmune disease, severe allergic disorder, malignancy, or infection.

This study was approved by the research ethics committee of the Keio University School of Medicine (#20140335) and was conducted according to the Declaration of Helsinki. Informed consent was obtained from all patients and HCs.

### Clinical assessment

Clinical information was obtained from patients’ records. We collected information on age; gender; time from symptom onset to diagnosis; body mass index at diagnosis; smoking habit; comorbidities, including hypertension, diabetes mellitus, dyslipidemia, chronic kidney disease, polymyalgia rheumatica (PMR) [13], and inflammatory bowel disease (IBD) [14]; laboratory data on erythrocyte sedimentation rate (ESR) and C-reactive protein (CRP) levels at diagnosis and each visit; and treatment during follow-up. Arterial involvement was evaluated using histological and/or radiological examinations (any or all of ultrasonography, computed tomography (CT), magnetic resonance imaging, and positron emission tomography CT).

Achievement of remission was defined as the disappearance of clinical symptoms with normal CRP with prednisolone (PSL) < 10 mg/day [5–10]. Relapse was defined as the reappearance of vasculitis-related manifestations accompanied by elevated levels of acute phase reactants requiring an increase in GC dose or additional immunosuppressive agents [5–10].

### FACS analysis

We collected clinical data and peripheral blood samples from patients with GCA and TAK at the time of diagnosis (week 0) and subsequently at weeks 4, 12, 24, and 52 of treatment. Twenty microliters of heparinized blood samples was collected from patients with LVV (GCA, *n* = 12; TAK, *n* = 8), and FACS analysis for immuno-phenotyping was carried out without delay after collecting the samples. FACS and data analyses were conducted on a FACS Aria II (BD Biosciences) and using FlowJo v.7.6.4 Software (Tree Star, Stanford University, CA, USA), according to the methods recommended by the manufacturers of the antibodies used (BD Biosciences and BioLegend: Additional file 4: Table S1). The phenotypes of immune cell subsets were defined based on the Human Immunology Project protocol [15]. Micro-sized cells, such as fragments of dead cells, were gated out when we analyzed the cells. Details of the gating strategy are shown in Additional file 4: Table S2 and Additional file 1: Figure S1. The mean number of each immune cell phenotype from patients with GCA and TAK was compared with that from age-matched HCs, and the fold change was calculated by dividing the cell number from patients by the corresponding average cell number from HCs. Fold change values for each immune cell phenotype were then compared between the groups.

Baseline data was analyzed using one-way analysis of variance (ANOVA) and post hoc test. Then, chronological data was analyzed using correlation analysis to identify the immune cell subsets associated with disease relapse. We also examined whether the use of biologic agents reduced the immune cells associated with disease relapse.

### Statistical analysis

Descriptive statistics were used to summarize the data. Continuous variables are shown as median and IQR. Baseline immuno-phenotyping data were analyzed by one-way ANOVA and post hoc test using the Tukey-Kramer test. The difference between pre- and post-treatment data was assessed using the Wilcoxon signed-rank test. The correlation coefficient was used for matrix correlation analysis. *p* values less than 0.05 were considered significant. All analyses were conducted using JMP version 14.0 (SAS Institute, Cary, NC, USA) or GraphPad Prism software V.8.0 (GraphPad, La Jolla, CA, USA).

## Results

### Baseline clinical characteristics and therapeutic response in LVV patients

We collected FACS data and clinical profiles from 20 Japanese LVV patients who were followed longitudinally across a total of 90 visits. None of the patients had been previously treated with GC or any biologic agent. All patients received GC therapy at an initial dose equivalent to 0.6–1.0 mg PSL/kg/day, which was tapered by the attending physician based on previously reported clinical trials [5–10].

We evaluated the patients at each visit based on their symptoms, ESR, CRP, and PSL dose. Baseline characteristics, treatment, and effects of treatment in patients with LVV and HCs are summarized in Table 1. TAK was younger (GCA vs TAK, 71 vs 47 years), and the time from onset to diagnosis was longer compared with GCA (2.5 vs 3.6 months). Fifty percent (6/12) of GCA were with PMR, and 25% (2/8) of TAK were with IBD. Laboratory tests of ESR and CRP were not different. The proportion of relapse and/or surgery was higher in TAK than in GCA (33% [4/12] vs 63% [5/8]).

**Table 1:** Clinical characteristics of patients with LVV

Variable	GCA, *n* = 12	TAK, *n* = 8	HC for GCA, *n* = 5	HC for TAK, *n* = 5
Baseline demographic				
Age at diagnosis, years (IQR)	71 (69–77)	47 (32–56)	72 (61–78)	50 (28–58)
Male, *n* (%)	7 (58)	4 (50)	2 (40)	1 (20)
Time from symptom onset to diagnosis, months (IQR)	2.5 (1.2–4.8)	3.6 (2.7–12)		
Body mass index at diagnosis, kg/m^2^ (IQR)	18 (17–22)	23 (17–24)		
Smoking, *n* (%)	6 (50)	3 (38)		
Comorbidities				
Hypertension, *n* (%)	4 (33)	1 (13)		
Diabetes mellitus, *n* (%)	2 (17)	0 (0)		
Dyslipidemia, *n* (%)	5 (42)	1 (13)		
Chronic kidney disease, *n* (%)	1 (8.3)	2 (25)		
PMR, *n* (%)	6 (50)	0 (0)		
IBD, *n* (%)	0 (0)	2 (25)		
Laboratory tests				
ESR, mm/h (IQR)	120 (115–134)	70 (56–109)		
CRP, mg/dL (IQR)	4.6 (2.2–8.2)	4.8 (2.1–7.9)		
Arterial involvement				
GCA: cranial/LV, *n*	8/4	–		
TAK: type I/IIa/IIb/III/IV/V, *n*	–	1/1/1/1/0/4		
Induction treatment				
Initial dose of PSL, mg (IQR)	50 (40–59)	60 (50–60)		
Biologic agents, *n* (%)	TCZ: 5 (42)	TCZ, 4 (50); IFX, 2 (25) (25)		
Relapse, *n* (%)	4 (33)	3 (38)		
Surgery, *n* (%)	0 (0)	3 (38)		
Relapse/surgery, *n* (%)	4 (33)	5 (63)		
Time to first relapse/surgery, months (IQR)	6.0 ± 4.0	3.6 ± 2.2		

*LVV* large vessel vasculitis, *GCA* giant cell arteritis, *TAK* Takayasu arteritis, *HC* healthy control, *PMR* polymyalgia rheumatica, *IBD* inflammatory bowel syndrome, *ESR* erythrocyte sedimentation rate, *CRP* C-reactive protein, *LV* large vessel, *PSL* prednisolone

### Baseline peripheral immune cell phenotypes in LVV patients

We compared the absolute number of circulating immune cells among GCA, TAK, and HCs using one-way ANOVA and post hoc test. We revealed that the number of helper T (Th), follicular helper T (Tfh), CD8^+^ T, CD8^+^ Tem, CD14^++^ CD16^+^ monocytes, and neutrophils was higher in patients with GCA and/or TAK than HCs. Among them, the number of CD8^+^ T and CD8^+^ Tem was higher in patients with TAK than GCA (Table 2).

**Table 2:** Comparison of peripheral immune cells among GCA, TAK, and HCs

Immune cell subtype (cells per μL)	GCA, *n* = 12	TAK, *n* = 8	HC for GCA, *n* = 5	HC for TAK, *n* = 5	ANOVA		Post hoc test	
					*p* value	GCA vs TAK	GCA vs HC	TAK vs HC
CD4^+^ T	491 ± 207	621 ± 282	512 ± 203	383 ± 231	0.36			
CD4 HLA-DR^+^	16 ± 8.9	11 ± 3.2	8.5 ± 0.80	7.6 ± 5.1	0.074			
CD4 naive	214 ± 38	248 ± 47	270 ± 66	210 ± 59	0.85			
CD4 Teff	5.1 ± 4.5	26 ± 53	5.4 ± 3.2	18 ± 21	0.46			
CD4 Tcm	194 ± 96	220 ± 90	143 ± 33	121 ± 71	0.18			
CD4 Tem	78 ± 49	128 ± 83	66 ± 1.0	77 ± 29	0.18			
Th1	80 ± 42	114 ± 18	62 ± 6.1	62 ± 30	0.23			
Th1 HLA-DR^+^	25 ± 14	18 ± 19	9.3 ± 3.1	13 ± 11	0.22			
Th2	40 ± 20	28 ± 18	20 ± 9.7	19 ± 18	0.10			
Th2 HLA-DR^+^	6.4 ± 1.6	3.0 ± 2.2	2.1 ± 1.0	3.1 ± 1.9	0.13			
Th17	57 ± 38	78 ± 33	36 ± 14	31 ± 14	0.061			
Th17 HLA-DR^+^	9.9 ± 8.5	10 ± 5.0	4.0 ± 1.9	5.1 ± 4.1	0.26			
Treg	20 ± 12	25 ± 16	11 ± 5.9	12 ± 5.3	0.16			
Treg HLA-DR^+^	8.9 ± 7.2	7.8 ± 4.2	5.0 ± 1.0	3.1 ± 1.5	0.20			
Tfh	68 ± 29	87 ± 31	63 ± 20	36 ± 21	*0.025*	0.43	0.98	*0.014*
Tfh1	16 ± 8.8	25 ± 3.3	15 ± 0.9	11 ± 4.9	0.063			
Tfh2	8.3 ± 5.0	9.1 ± 8.8	4.1 ± 1.1	5.2 ± 3.6	0.41			
Tfh17	24 ± 11	29 ± 9.8	23 ± 10	10 ± 7.6	*0.035*	0.21	0.99	0.057
Tfr	5.1 ± 2.5	5.6 ± 3.5	3.7 ± 0.14	2.6 ± 2.6	0.23			
CD8^+^ T	189 ± 115	331 ± 128	228 ± 126	163 ± 52	*0.039*	*0.044*	0.93	*0.033*
CD8 HLA-DR^+^	24 ± 22	16 ± 9.6	5.6 ± 2.6	6.6 ± 4.1	0.12			
CD8 naive	23 ± 17	77 ± 50	94 ± 61	74 ± 19	*0.015*	0.054	*0.043*	0.99
CD8 Teff	90 ± 89	98 ± 52	30 ± 12	43 ± 23	0.24			
CD8 Tcm	20 ± 13	28 ± 21	9.9 ± 4.6	12 ± 6.6	0.15			
CD8 Tem	57 ± 35	128 ± 87	54 ± 23	51 ± 15	*0.024*	*0.028*	0.99	*0.033*
γδT	5.5 ± 5.1	22 ± 26	4.7 ± 1.7	18 ± 32	0.22			
NKT	29 ± 33	43 ± 34	3.7 ± 0.7	29 ± 13	0.22			
CD19^+^ B	97 ± 51	211 ± 175	226 ± 89	94 ± 42	*0.045*	0.11	0.17	0.23
CD27^−^ IgD^+^ naive B	79 ± 48	161 ± 151	186 ± 77	52 ± 13	0.052			
CD27^−^ IgD^+^ activated B	2.4 ± 1.6	6.7 ± 5.3	13 ± 4.8	13 ± 11	*0.0038*	0.35	*0.017*	0.29
CD27^+^ IgD^−^ memory B	11 ± 5.8	31 ± 20	18 ± 3.9	16 ± 12	*0.015*	*0.0086*	0.74	0.17
Plasmablast	0.6 ± 0.8	3.3 ± 5.3	0.2 ± 0.06	0.9 ± 1.0	0.17			
Plasma cell	0.5 ± 0.5	2.1 ± 3.4	0.05 ± 0.0	0.3 ± 0.4	0.17			
NK	171 ± 170	149 ± 77	123 ± 6.4	153 ± 48	0.92			
DC	14 ± 9.7	17 ± 14	21 ± 3.7	19 ± 15	0.72			
mDC	8.2 ± 5.8	6.6 ± 2.3	15 ± 1.4	11 ± 5.4	*0.035*	0.88	0.071	0.43
pDC	1.3 ± 1.0	2.2 ± 1.4	3.2 ± 1.7	4.5 ± 6.4	0.20			
Monocyte	209 ± 91	258 ± 30	93 ± 43	158 ± 38	*0.024*	0.59	0.11	*0.020*
CD14^++^ CD16^−^	144 ± 73	188 ± 94	80 ± 4.3	116 ± 35	0.096			
CD14^++^ CD16^+^	48 ± 36	44 ± 22	5.3 ± 0.5	20 ± 15	*0.040*	0.98	*0.049*	0.30
CD14^+^ CD16^+^	11 ± 7.0	16 ± 7.3	4.5 ± 3.5	12 ± 3.1	0.084			
Eosinophil	76 ± 36	135 ± 115	50 ± 40	63 ± 45	0.14			
Neutrophil	4009 ± 2054	4329 ± 2425	1232 ± 688	1551 ± 759	*0.013*	0.98	0.073	*0.036*
Basophil	30 ± 25	35 ± 14	30 ± 0.1	26 ± 26	0.90			

Comparison between of baseline peripheral immune cell number between GCA (*n* = 12), TAK (n = 8), HC for GCA (*n* = 5), and HC for TAK (*n* = 5) using ANOVA and post hoc test

*Th* helper T, *Tfh* follicular helper T, *NK* natural killer, *DC* dendritic cell, *mDC* myeloid DC, *pDC* plasmacytoid DC

**Fig. 1:**
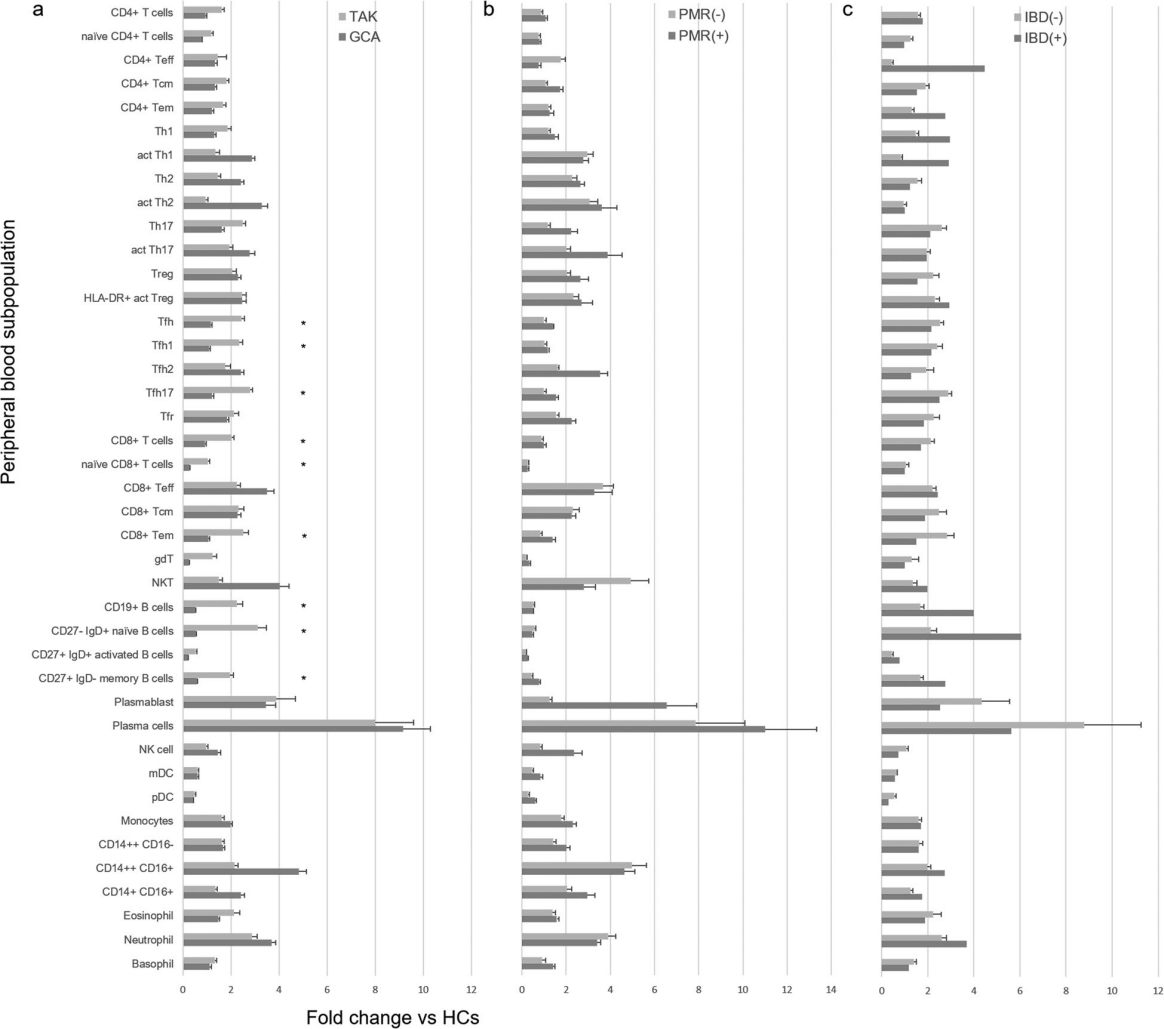
Fold change in each immuno-phenotype in patients with LVV. Fold change in each immuno-phenotype at baseline in patients with **a** GCA (*n* = 12) and TAK (*n* = 8), **b** GCA with PMR (*n* = 6) and without PMR (*n* = 6), and **c** TAK with IBD (*n* = 2) and without IBD (*n* = 6) was compared with the mean number of corresponding immune cells in healthy controls. LVV, large vessel vasculitis; GCA, giant cell arteritis; TAK, Takayasu arteritis; PMR, polymyalgia rheumatica; IBD, inflammatory bowel disease; HCs, healthy controls. Data are shown as mean ± SEM. *p < 0.05 for analysis using Mann-Whitney *U* test

Fold change in the number of immune cells in patients with GCA and TAK was determined by dividing the cell number in patients by the average cell number of the corresponding immuno-phenotype in age-matched HCs (Fig. 1). Then, fold changes of each cell subset in patients with GCA or TAK, GCA with or without PMR, and TAK with or without IBD were compared. The proportion of Tfh, Tfh1, Tfh17, CD8^+^ T, naive CD8^+^ T, CD8^+^ Tem, CD19^+^ B, CD27^−^ IgD^+^ naive B, and CD27^+^ IgD^−^ memory B was higher in TAK than in GCA, which was consistent with ANOVA (Fig. 1a). There was no statistical difference in the proportion of cell subsets between patients with or without PMR/IBD (Fig. 1b, c).

### Chronological changes in immuno-phenotype associated in LVV without relapse

**Fig. 2:**
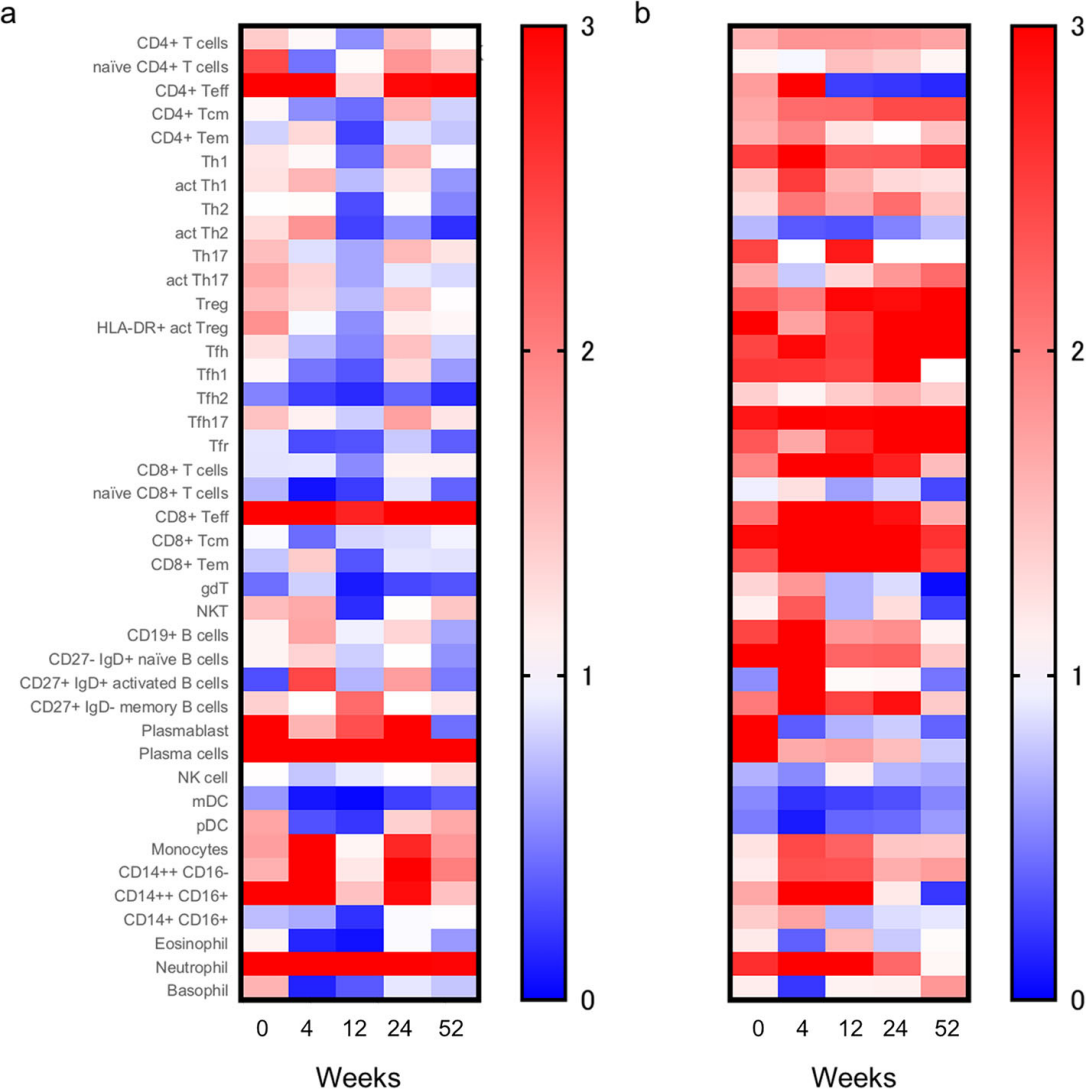
Heatmap of baseline and time-course immuno-phenotyping in each subset in LVV patients without relapse. Fold change in each immuno-phenotype at baseline and at weeks 4, 12, 24, and 52 of treatment in **a** GCA (*n* = 8) and **b** TAK (*n* = 5) patients without relapse. LVV, large vessel vasculitis; GCA, giant cell arteritis; TAK, Takayasu arteritis

Because of the higher proportion of relapse/surgery in patients with TAK than GCA [3, 16, 17], we hypothesized that the immuno-phenotype profile may differ after treatment between the two groups of the patients. Thus, we compared the immuno-phenotyping data between GCA (*n* = 8) and TAK (*n* = 5) patients without relapse. Compared to patients with GCA (Fig. 2a), memory CD4^+^ T and CD8^+^ T cells in patients with TAK remained high even in the remission phase (Fig. 2b). To assess the effect of treatments, we analyzed the fold changes in immune cell subsets in patients successfully treated with GC and biologic agents (GCA, *n* = 5; TAK, *n* = 3). As a result, we found that memory CD4^+^ T and CD8^+^ T cells in patients with TAK also remained high levels even after treatment with biologic agents (Additional file 2: Figure S2A and S2B).

### Chronological changes in immuno-phenotype associated with disease relapse

To assess changes in immuno-phenotype at relapse, we longitudinally profiled the immuno-phenotyping data of patients who achieved remission then relapsed. We followed the immuno-phenotyping data of seven patients (GCA, *n* = 4; TAK, *n* = 3) who achieved remission then relapsed until week 52 of treatment and calculated the correlation between the fluctuation in each immune cell subset and change in disease activity.

**Fig. 3:**
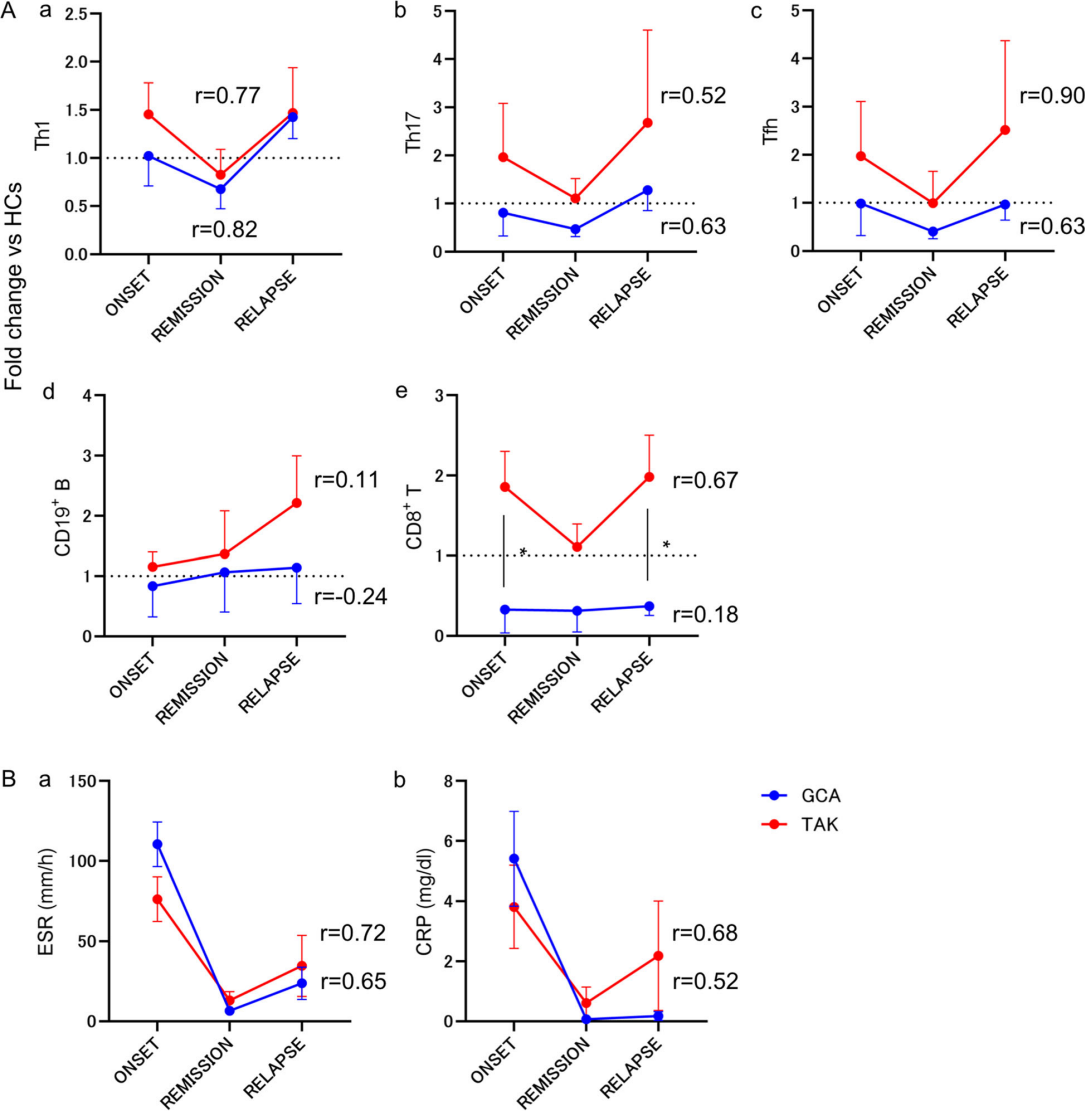
Changes in the proportion of selected immune cells and laboratory findings along with disease activity in LVV patients with relapse. Changes in the fold change of selected immune cells and laboratory findings along with the disease activity were shown. The correlation score was calculated using correlation matrix analysis. **A** The fold changes of (a) Th1, (b) Th17, (c) Tfh, (d) CD19^+^ B, and (e) CD8^+^ cells and **B** laboratory data of (a) ESR and (b) CRP in LVV patients with disease relapse (GCA, *n* = 4; TAK, *n* = 3). LVV, large vessel vasculitis; GCA, giant cell arteritis; TAK, Takayasu arteritis; Th, helper T; Tfh, follicular helper T; ESR, erythrocyte sedimentation rate; CRP, C-reactive protein. Dotted lines showed healthy controls. Data are shown as mean ± SD. *p < 0.05 for Mann-Whitney *U* test

The results of matrix correlation analysis for each subset and the fluctuation in disease activity are shown in Additional file 3: Figure S3. Given that Th1 and Th17 have been implicated in the pathogenesis of GCA and TAK [18–20], we examined the changes in relevant selected subsets including Th1, Th17, Tfh, CD8^+^ T, and CD19^+^ B cells and laboratory data of ESR and CRP (Fig. 3). Th1, Th17, and Tfh cells were strongly correlated with disease activity in GCA and TAK (Fig. 3A), as well as ESR and CRP levels (Fig. 3B). Notably, CD8^+^ T cells were strongly correlated with disease activity in TAK, but not in GCA. In addition, the fold changes of CD8^+^ T cells at onset (TAK vs GCA, 1.9 ± 0.8 vs 0.3 ± 0.3, *p* = 0.032) and at relapse (2.0 ± 0.9 vs 0.4 ± 0.1, *p* = 0.011) were significantly higher in patients with TAK than those in GCA.

### Chronological changes in immuno-phenotype associated with treatment with biologics

**Fig. 4:**
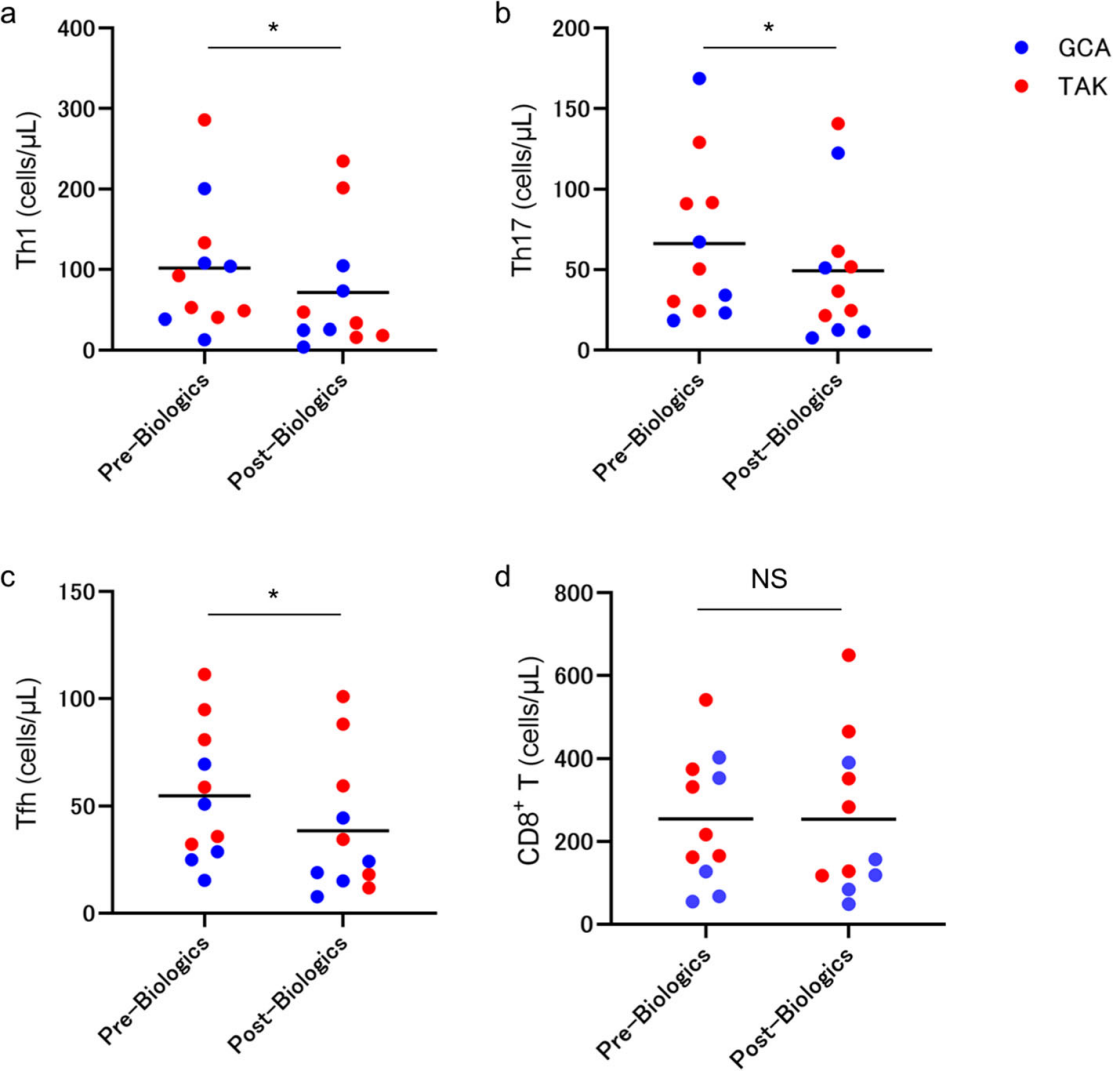
The number of selected immune cell subsets before and after treatment with biologic agents. The number of **a** Th1, **b** Th17, **c** Tfh, and **d** CD8^+^ T cells before and after treatment with biologic agents in GCA (*n* = 5) and TAK (*n* = 6). Th, helper T; Tfh, follicular helper T; GCA, giant cell arteritis; TAK, Takayasu arteritis. *p < 0.05 for Wilcoxon signed-rank test. NS, not significant

Eleven patients (GCA, *n* = 5; TAK: *n* = 6) were treated with TCZ (*n* = 9) and IFX (*n* = 2). To determine whether these drugs cause immunological changes, we compared the number of immune cells of selected subsets before and after treatment with biologic agents (Fig. 4). It is noteworthy that treatment with biologic agents did not decrease the proportion of CD8^+^ T cells (Fig. 4d), while the treatment decreased the proportion of Th1, Th17, and Tfh cells (Fig. 4a–c).

## Discussion

This study examined the baseline and chronological changes in immuno-phenotype profiles in patients with LVV. We identified the common characteristics and differences in immuno-phenotypes between patients with GCA and TAK. CD8^+^ T and CD8^+^ Tem were higher in TAK patients than in GCA or HCs. Further study using matrix correlation analysis revealed that Th1, Th17, Tfh, and CD8^+^ T cells were associated with disease relapse in TAK. Changes in these immune cells were useful for distinguishing active LVV from remission. We also revealed that treatment with biologic agents decreased the number of Th1, Th17, and Tfh cells, suggesting that suppression of these immune cells may be responsible for the drugs’ therapeutic effects.

Compared to the immuno-phenotypes in HCs, the number of Th, Tfh, CD8^+^ T, CD14^++^ CD16^+^ intermediate monocytes, and neutrophils were elevated in patients with GCA and/or TAK. A recent report by our group also found that similar subsets of immune cells were elevated in patients with anti-neutrophil cytoplasmic antibody-associated vasculitis [21]. CD14^++^ CD16^+^ monocytes and neutrophils may be involved in a common pathway with important roles in small to large vessel vasculitis. Tertiary lymphoid organs are typically identified using arterial histological examination [22], which may explain the increase in Th and Tfh cells in LVV patients.

Comparison of immuno-phenotypes in patients with GCA and TAK demonstrated that the number of CD8^+^ T cells at onset and at relapse was higher in patients with TAK than in those with GCA. TAK is associated with major histocompatibility (MHC) class I alleles, human leukocyte antigen (HLA)-A24 and B52 [23, 24], while GCA is associated with MHC class II alleles, HLA-DR4 [25, 26], suggesting that TAK may be associated with activation of CD8^+^ T cells. Additionally, a comparison between remission and non-remission phases showed that the proportion of immune cells of several subsets remained elevated in TAK patients who had achieved remission, suggesting that conventional clinical assessments to evaluate disease activity in TAK are limited. Despite considerable discussion on the potential differences between GCA and TAK [3, 16–20, 27], the immunological difference remains unknown. The higher relapse rate in patients with TAK compared to those with GCA during treatment is consistent with our finding that immune cell involvement is persistently present in patients with TAK.

GC is the mainstay of treatment for LVV, while conventional immunosuppressants have modest GC-sparing effects. Recent studies have shown that biological drugs may be a valid therapeutic option, especially in patients with severe and/or relapsing LVV. A previous report showed that regulatory T cells are increased after treatment with TCZ [28]. Despite this, we do not currently have a complete picture of how immune cell profiles reflect treatment with biologic agents. We found that the number of Th1, Th17, Tfh, and CD8^+^ T cells was correlated with disease activity and may therefore be useful alternative biomarkers for evaluating disease status. TCZ and IFX decreased the number of Th1, Th17, and Tfh cells, suggesting that these biologic agents exert their effects by regulating the function of these pathogenic immune cells. In contrast, neither TCZ nor IFX suppressed CD8^+^ T cells, which may play an important role in the pathogenesis of TAK. New treatments targeting CD8^+^ T cells are needed.

Our study has several limitations. First, this is a study with a small sample size and with a short observation period. Second, we did not investigate the types of immune cells that infiltrated the affected tissues. Further analysis with a large cohort may be required to clarify the responsible cell subsets of the pathogenesis of LVV.

Allowing for these limitations, this is the first study to comprehensively demonstrate the peripheral immuno-phenotype profile of LVV. We demonstrated the differences of the baseline and time-course immuno-phenotype profiles between GCA and TAK.

## Conclusions

Our results from peripheral immuno-phenotyping analysis indicate that the numbers of Th and Tfh cells changed along with the disease condition in both GCA and TAK, while that of CD8^+^ T cells did not, especially in TAK. Chronological immuno-phenotyping data explained the difference in therapeutic response, such as reactivities against biologics, between GCA and TAK. Accumulation of further evidence on immuno-phenotype profiles is expected to improve treatment options for LVV patients.

## Supplementary information

**Additional file 1 : Figure S1** Immuno-phenotyping strategy using antibody staining.

**Additional file 2: Figure S2** Heatmap of baseline and time-course immuno-phenotyping in patients successfully treated with GC and biologic agents. Fold change in each immuno-phenotype at baseline and at weeks 4, 12, 24 and 52 of treatment with GC and biologic agents in (A) GCA (*n*=5) and (B) TAK (*n*=3) patients without relapse. GC: glucocorticoid, GCA: giant cell arteritis, TAK: Takayasu arteritis.

**Additional file 3 : Figure S3** Correlation analysis of disease activity with the number of immune cells in LVV patients with relapse. Correlation coefficient for the number of each immune cells of each subset and disease activity in GCA (*n*=4) and TAK (n=3) patients with relapse. LVV: large vessel vasculitis, GCA: giant cell arteritis, TAK: Takayasu arteritis.

**Additional file 4 :Table S1**. Antibodies used in FACS analysis. **Table S2**. Immuno-phenotyping strategy using antibody staining.

## Data Availability

All data generated or analyzed during this study are included in this published article and its supplementary information files.

## Abbreviations

CRP: C-reactive protein
CT: Computed tomography
ESR: Erythrocyte sedimentation rate
GC: Glucocorticoid
GCA: Giant cell arteritis
HCs: Healthy controls
HLA: Human leukocyte antigen
IBD: Inflammatory bowel disease
IFX: Infliximab
IL: Interleukin
LVV: Large vessel vasculitis
MHC: Major histocompatibility
PMR: Polymyalgia rheumatica
PSL: Plednisolone
TAK: Takayasu arteritis
TCZ: Tocilizumab
Tfh: Follicular helper T
Th: Helper T
TNF: Tumor necrosis factor
Treg: Regulatory T
