# Supplementary figures and images for: Significant association between clinical characteristics and changes in peripheral immuno-phenotype in large vessel vasculitis
Source: Arthritis Res Ther. 2019 Dec 30;21:304. doi: 10.1186/s13075-019-2068-7 (PMC6937853; doi:10.1186/s13075-019-2068-7)

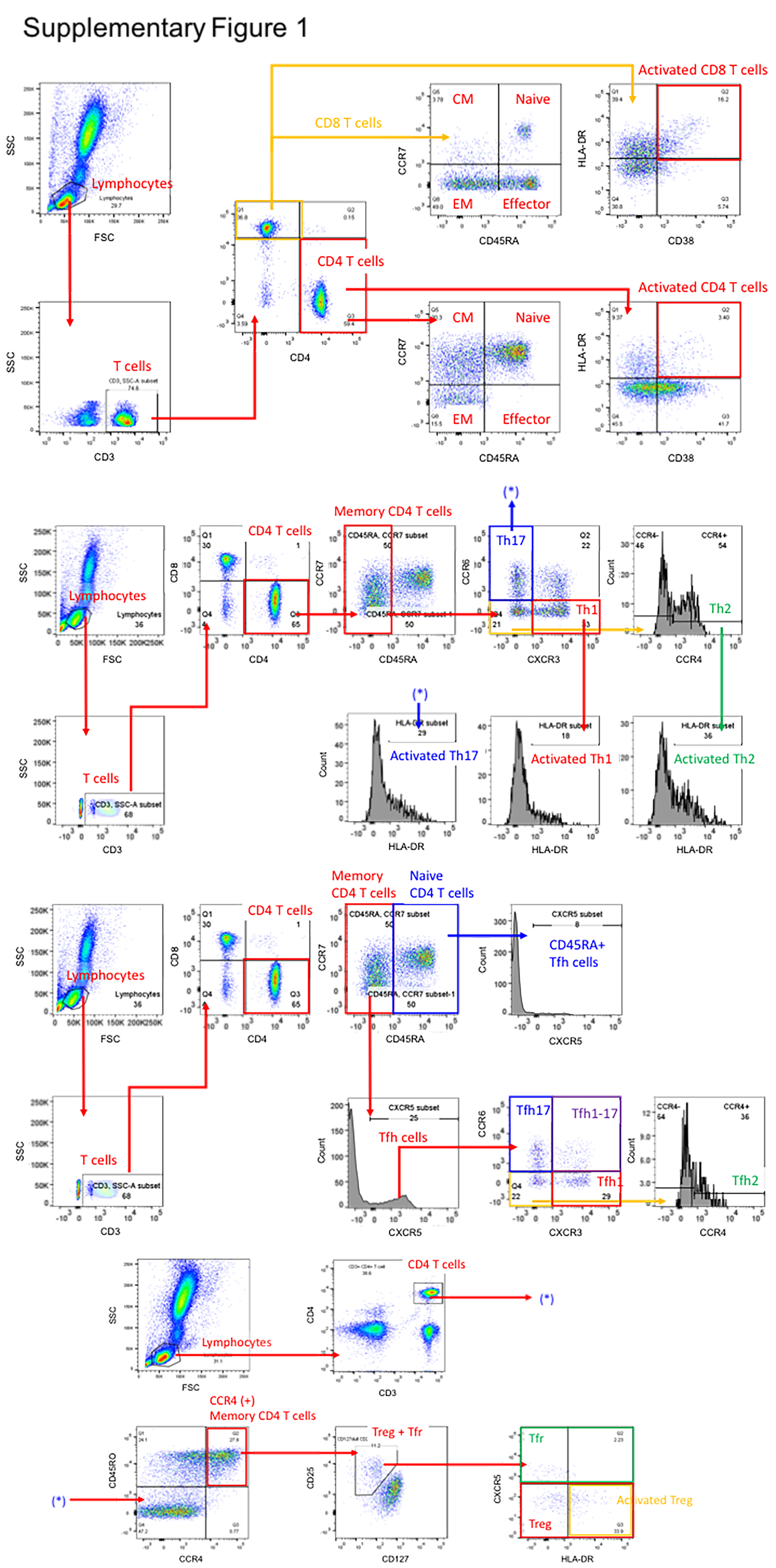

Supplement: Supplementary file 1 — Additional file 1 : Figure S1 Immuno-phenotyping strategy using antibody staining. [file 13075_2019_2068_MOESM1_ESM.zip › Supplementary Fig.1(1)_600.tif]

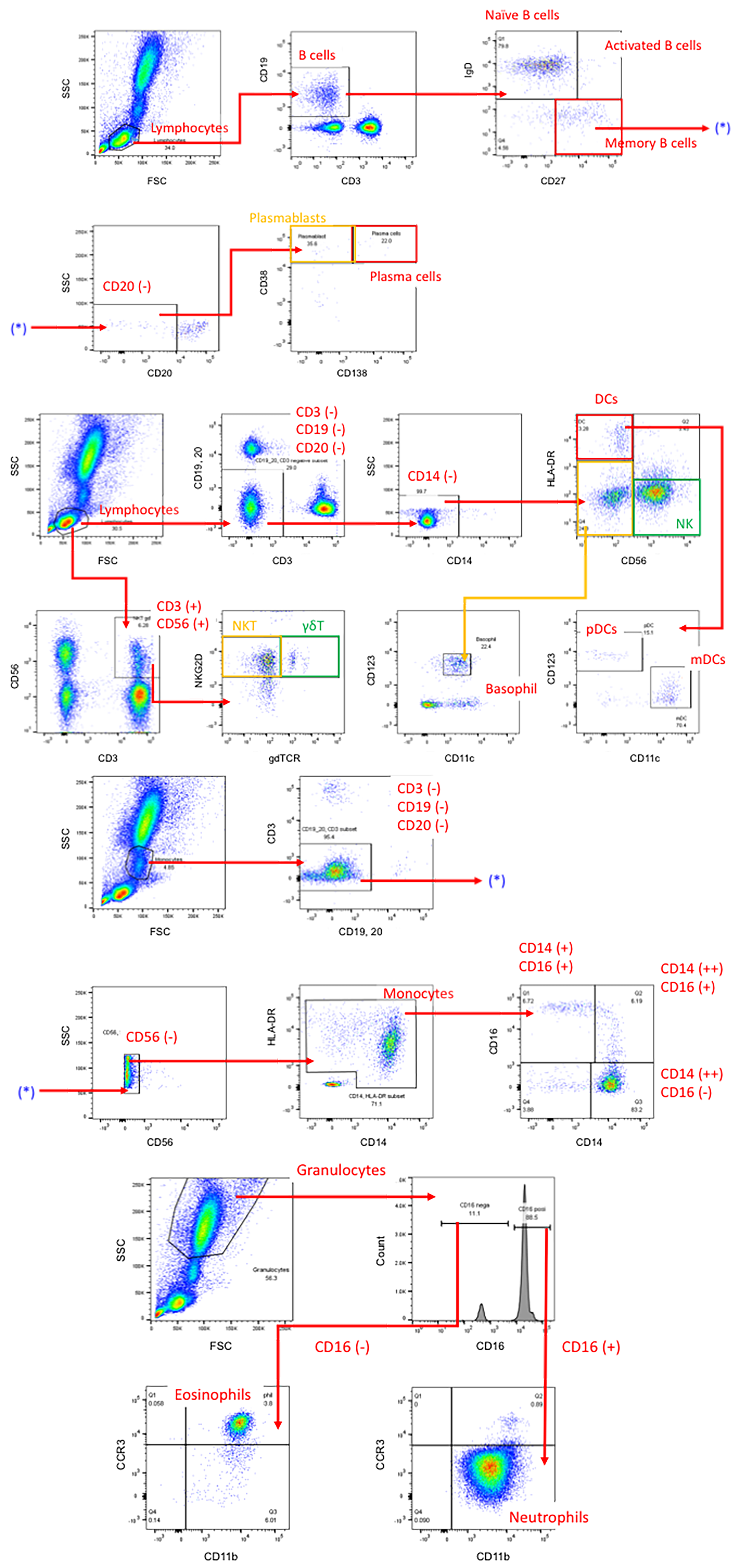

Supplement: Supplementary file 1 — Additional file 1 : Figure S1 Immuno-phenotyping strategy using antibody staining. [file 13075_2019_2068_MOESM1_ESM.zip › Supplementary Fig.1(2)_600.tif]

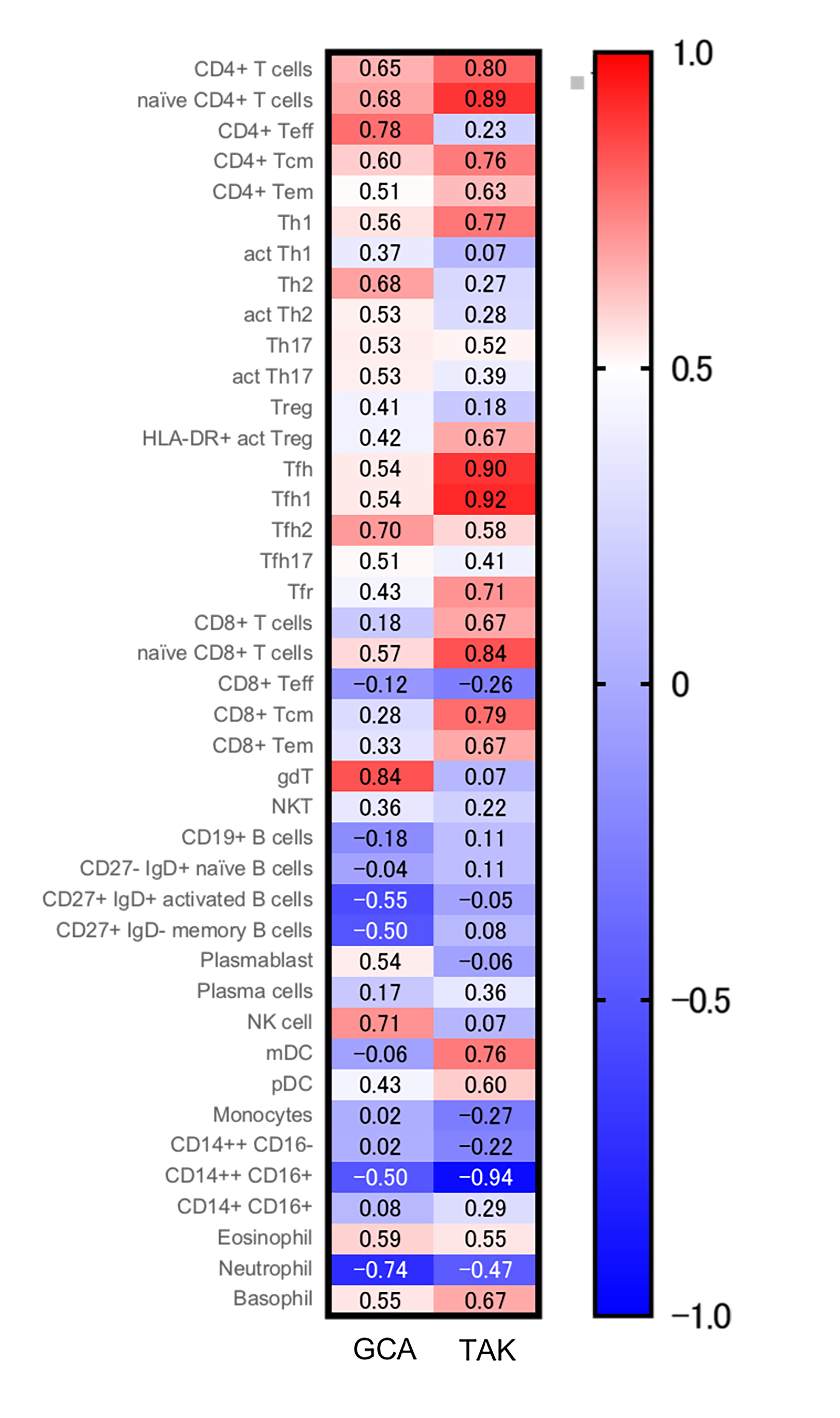

Supplement: Supplementary file 3 — Additional file 3 : Figure S3 Correlation analysis of disease activity with the number of immune cells in LVV patients with relapse. Correlation coefficient for the number of each immune cells of each subset and disease activity in GCA (n=4) and TAK (n=3) patients with relapse. LVV: large vessel vasculitis, GCA: giant cell arteritis, TAK: Takayasu arteritis. [file 13075_2019_2068_MOESM3_ESM.tif]
