# Supplementary material for: Significant association between clinical characteristics and changes in peripheral immuno-phenotype in large vessel vasculitis
Source: Arthritis Res Ther. 2019 Dec 30;21:304. doi: 10.1186/s13075-019-2068-7 (PMC6937853; doi:10.1186/s13075-019-2068-7)
Supplement: Supplementary file 4 — Additional file 4 :Table S1. Antibodies used in FACS analysis. Table S2. Immuno-phenotyping strategy using antibody staining. [file 13075_2019_2068_MOESM4_ESM.docx]

Table S1. Antibodies used in FACS analysis.

| Anti-human surface antigen antibody | Fluorecent dye | Clone |
| --- | --- | --- |
| CD2 | BV605 | RPA-2.10 |
| CD3 | FITC | UCHT1 |
| CD3 | PE-CF594 | UCHT1 |
| CD3 | BV786 | SK7 |
| CD4 | BV786 | SK3 |
| CD8 | APC-Cy7 | RPA-T8 |
| CD8 | BV711 | RPA-T8 |
| CD11b | BV711 | ICRF44 |
| CD11c | PE-Cy7 | B-ly6 |
| CD14 | BV605 | M5E2 |
| CD16 | APC | 3G8 |
| CD19 | PerCP-Cy5.5 | HIB19 |
| CD19 | APC-H7 | HIB19 |
| CD20 | APC-H7 | 2H7 |
| CD24 | BV421 | ML5 |
| CD25 | PE | M-A251 |
| CD27 | PE-Cy7 | M-T271 |
| CD38 | APC | HIT2 |
| CD45RA | PE-Cy7 | HI100 |
| CD45RO | APC-Cy7 | UCHL1 |
| CD56 | FITC | B159 |
| CD123 | PerCP-Cy5.5 | 7G3 |
| CD127 | FITC | HIL-7R-M21 |
| CD138 | BV605 | MI15 |
| CD183 | PE | 1C6/CXCR3 |
| CD193 (CCR3) | PE/Dazzle 594 | 5E8 |
| CD194 | PE-Cy7 | 1G1 |
| CD194 | BV605 | 1G1 |
| CD196 | BV711 | G034E3 |
| CD197 (CCR7) | PE-CF594 | 150503 |
| CD279 (PD-1) | BV510 | EH12.1 |
| CD314 (NKG2D) | PE | 1D11 |
| CXCR5 | BV421 | RF8B2 |
| HLA-DR | PE-CF594 | G46-6 |
| HLA-DR | PerCP-Cy5.5 | G46-6 |
| HLA-DR | BV510 | L243 |
| IgD | BV510 | IA6-2 |
| TCR α/β | BV510 | IP26 |
| TCR γδ | BV421 | B1 |

Table S2. Immuno-phenotyping strategy using antibody staining.

| Immune cell subtype |  |
| --- | --- |
| **CD4+ T** | CD3+ CD4+ CD8- |
| CD4 HLA-DR+ | CD3+ CD4+ CD8- CD38+ HLA-DR+ |
| CD4 naïve | CD3+ CD4+ CD8- CCR7+ CD45RA+ |
| CD4 Teff | CD3+ CD4+ CD8- CCR7- CD45RA+ |
| CD4 Tcm | CD3+ CD4+ CD8- CCR7+ CD45RA- |
| CD4 Tem | CD3+ CD4+ CD8- CCR7- CD45RA- |
| Th1 | CD3+ CD4+ CD8- CD45RA- CCR6- CXCR3+ |
| Th1 HLA-DR+ | CD3+ CD4+ CD8- CD45RA- CCR6- CXCR3+ HLA-DR+ |
| Th2 | CD3+ CD4+ CD8- CD45RA- CCR6- CXCR3- |
| Th2 HLA-DR+ | CD3+ CD4+ CD8- CD45RA- CCR6- CXCR3- HLA-DR+ |
| Th17 | CD3+ CD4+ CD8- CD45RA- CCR6+ CXCR3- |
| Th17 HLA-DR+ | CD3+ CD4+ CD8- CD45RA- CCR6+ CXCR3- HLA-DR+ |
| Treg | CD3+ CD4+ CD45RO+ CCR4+ CD25+ CD127- CXCR5- |
| Treg HLA-DR+ | CD3+ CD4+ CD45RO+ CCR4+ CD25+ CD127- CXCR5- HLA-DR+ |
| Tfh naïve | CD3+ CD4+ CD8- CD45RA+ CXCR5+ |
| Tfh | CD3+ CD4+ CD8- CD45RA- CXCR5+ |
| Tfh1 | CD3+ CD4+ CD8- CD45RA- CXCR5+ CCR6- CXCR3+ |
| Tfh2 | CD3+ CD4+ CD8- CD45RA- CXCR5+ CCR6- CXCR3- |
| Tfh17 | CD3+ CD4+ CD8- CD45RA- CXCR5+ CCR6+ CXCR3- |
| Tfr | CD3+ CD4+ CD45RO+ CCR4+ CD25+ CD127- CXCR5+ |
| **CD8+ T** | CD3+ CD4- CD8+ |
| CD8 HLA-DR+ | CD3+ CD4- CD8+ CD38+ HLA-DR+ |
| CD8 naïve | CD3+ CD4- CD8+ CCR7+ CD45RA+ |
| CD8 Teff | CD3+ CD4- CD8+ CCR7- CD45RA+ |
| CD8 Tcm | CD3+ CD4- CD8+ CCR7+ CD45RA- |
| CD8 Tem | CD3+ CD4- CD8+ CCR7- CD45RA- |
| **γδT** | CD3+ CD56+ NKG2D+ γδTCR+ |
| **NKT** | CD3+ CD56+ NKG2D+ γδTCR- |
| **CD19+ B** | CD3- CD19+ |
| CD27- IgD+ naïve B | CD3- CD19+ CD27- IgD+ |
| CD27+ IgD+ activated B | CD3- CD19+ CD27+ IgD+ |
| CD27+ IgD- memory B | CD3- CD19+ CD27- IgD- |
| Plasmablast | CD3- CD19+ CD27- IgD- CD20- CD38+ CD138- |
| Plasma cell | CD3- CD19+ CD27- IgD- CD20- CD38+ CD138+ |
| **NK** | CD3- CD19- CD20- CD14- CD56+ HLA-DR- |
| **DC** | CD3- CD19- CD20- CD14- CD56- HLA-DR+ |
| mDC | CD3- CD19- CD20- CD14- CD56- HLA-DR+ CD11c- CD123+ |
| pDC | CD3- CD19- CD20- CD14- CD56- HLA-DR+ CD11c+ CD123- |
| **Monocyte** | CD3- CD19- CD20- HLA-DR+ |
| CD14++ CD16- | CD14++ CD16- |
| CD14++ CD16+ | CD14++ CD16+ |
| CD14+ CD16+ | CD14+ CD16+ |
| **Eosinophil** | CD16- CD11b+ CCR3+ |
| **Neutrophil** | CD16+ CD11b+ CCR3- |
| **Basophil** | CD3- CD19- CD20- CD14- CD56- HLA-DR- CD11c+ CD123+ |

Th: helper T, Tfh: follicular helper T, NK: natural killer, DC: dendritic cell, mDC: myeloid DC, pDC: plasmacytoid DC.
